# Modeling the distribution of the West Nile and Rift Valley Fever vector *Culex pipiens* in arid and semi-arid regions of the Middle East and North Africa

**DOI:** 10.1186/1756-3305-7-289

**Published:** 2014-06-24

**Authors:** Amy K Conley, Douglas O Fuller, Nabil Haddad, Ali N Hassan, Adel M Gad, John C Beier

**Affiliations:** 1 Department of Geography, University of Miami, 1300 Campo Sano Avenue, Coral Gables, FL 33146, USA; 2 College of Arts and Sciences, University of Miami, Miami, FL, USA; 3 Laboratory of Immunology, Faculty of Public Health, Lebanese University, Fanar, El-Metn, Lebanon; 4 Department of Basic Environmental Sciences, Institute of Environmental Studies & Research, Ain Shams University, Cairo, Egypt; 5 Entomology Department, Ain Shams University, Cairo, Egypt; 6 Department of Public Health Sciences, University of Miami Miller School of Medicine, Miami, FL, USA

**Keywords:** Species distribution models, Remote sensing, *Culex pipiens*, Maxent, Public health, Arid region, Irrigation

## Abstract

**Background:**

The Middle East North Africa (MENA) region is under continuous threat of the re-emergence of West Nile virus (WNV) and Rift Valley Fever virus (RVF), two pathogens transmitted by the vector species *Culex pipiens.* Predicting areas at high risk for disease transmission requires an accurate model of vector distribution, however, most *Cx. pipiens* distribution modeling has been confined to temperate, forested habitats. Modeling species distributions across a heterogeneous landscape structure requires a flexible modeling method to capture variation in mosquito response to predictors as well as occurrence data points taken from a sufficient range of habitat types.

**Methods:**

We used presence-only data from Egypt and Lebanon to model the population distribution of *Cx. pipiens* across a portion of the MENA that also encompasses Jordan, Syria, and Israel. Models were created with a set of environmental predictors including bioclimatic data, human population density, hydrological data, and vegetation indices, and built using maximum entropy (Maxent) and boosted regression tree (BRT) methods. Models were created with and without the inclusion of human population density.

**Results:**

Predictions of Maxent and BRT models were strongly correlated in habitats with high probability of occurrence (Pearson’s r = 0.774, r = 0.734), and more moderately correlated when predicting into regions that exceeded the range of the training data (r = 0.666,r = 0.558). All models agreed in predicting high probability of occupancy around major urban areas, along the banks of the Nile, the valleys of Israel, Lebanon, and Jordan, and southwestern Saudi Arabia. The most powerful predictors of *Cx. pipiens* habitat were human population density (60.6% Maxent models, 34.9% BRT models) and the seasonality of the enhanced vegetation index (EVI) (44.7% Maxent, 16.3% BRT). Maxent models tended to be dominated by a single predictor. Areas of high probability corresponded with sites of independent surveys or previous disease outbreaks.

**Conclusions:**

*Cx. pipiens* occurrence was positively associated with areas of high human population density and consistent vegetation cover, but was not significantly driven by temperature and rainfall, suggesting human-induced habitat change such as irrigation and urban infrastructure has a greater influence on vector distribution in this region than in temperate zones.

## Background

With rapidly expanding urban populations, recent refugee movements, internal displacement, and widespread civil strife, parts of the Middle East are becoming increasingly vulnerable to vector-borne diseases (VBDs). Urban growth, in particular, has expanded faster than the supply of available housing and supporting infrastructure, resulting in a large proportion of the population living in unsanitary conditions, which may increase human exposure to bites of infected vectors that proliferate in urban environments [1-4].

Prior to the recent period of civil conflict, the region has experienced the emergence and resurgences of two deadly diseases. Rift Valley Fever Virus caused an epidemic in Egypt in 1977, 1993 and 2003, and hit Saudi Arabia and Yemen in 2000 and 2001 [5-7]. In addition, West Nile virus, a nearly “forgotten” disease re-emerged in a severe country-wide epidemic in Israel in 2000, with a fatality rate of 8.4% [8]. Between 2010 and 2012, 200 West Nile cases were reported in Israel [9]. While the causes for the recent resurgence of neglected tropical diseases like West Nile Virus, Rift Valley Fever Virus, and Leishmaniasis are not fully understood, it is cause for renewed interest and increased attention to disease dynamics in the region [2,10,11].

Molecular and epidemiological studies have shown that *Cx. pipiens* is a vector of both Rift Valley Fever virus and West Nile Virus [6,12-16]. It has a preference for living near areas of high human population density, and breeding in the artificial containers abundant in close proximity to human settlements [17,18] or polluted pools of water associated with human activities [19-22]. The species also feeds opportunistically from a wide variety of blood hosts [23,24], and is active nearly year round [21]. This vast ecological plasticity makes it a potentially significant vector. As such, understanding the distribution of *Cx. pipiens* throughout the region is a fundamental requirement to understanding transmission dynamics.

Habitat suitability models for *Cx. pipiens* in general have identified rainfall, temperature, and vegetation to be major drivers of population distribution [25,26]. Density of hosts has also been found to be significantly correlated with habitat suitability in related species [27]. However, these studies were largely conducted in different climatic regions than those dominant in the MENA region, and therefore the identified predictors would not necessarily explain vector distributions in arid and semi-arid regions. Environmental predictors will be most informative at scales and across regions where they exhibit sufficient heterogeneity to accurately discern suitable from unsuitable habitat. The relative information values of predictors shift as underlying climatic conditions change, and so does the nature and identity of the most significant vector-habitat relationship in the model. In Egypt, climatic variables such as ambient temperature, relative humidity, and wind speed did not significantly distinguish sites with high and low filariasis transmission vectored by *Cx. pipiens,* which was positively correlated with temperature and negatively correlated with rainfall [28]*.* In Australia, the critical climate factors for predicting outbreaks of Ross River virus vary significantly between different environmental regions within a single state [29]. Simulation models of *Cx. quinquefasciatus* across the southern United States revealed significantly different sensitivities of mosquito populations to temperature and precipitation in arid and humid habitats [30].

The aim of the present work is to investigate the relationship between the distribution of *Cx. pipiens* and the environment in the Middle East and subsequently derive vector distribution surfaces. Accordingly, we used data collected from a wide range of habitats in the region and two different but robust presence-only species distribution models (SDMs), Maxent and boosted regression trees (BRT). The models examined the role of human populations, climatic, hydrological, and vegetation parameters in predicting habitat suitability.

## Methods

### Selecting modeling approaches

In order to strengthen confidence in predicting *Cx. pipiens* distributions, we incorporated two modeling approaches, Maximum entropy implemented in Maxent software, and boosted regression trees. Regression models are powerful tools for selecting relevant predictors and modeling complex interactions, while boosting avoids misclassification problems inherent in a single tree model. Thus our combined models would enable powerful selection of relevant predictors and accurate modeling of complex interactions. The previously discussed non-uniform relationships between other Culex species and critical climate factors [30] suggest such interactions may possibly contribute significantly to *Cx. pipiens* distributions as well. Thus, the use of model intercomparisons leads to greater confidence in the predictions of the distribution. Using Maxent as our alternative method allows for comparisons with the predictions of one of the most familiar and frequently applied presence only modeling techniques [31-34].

### Modeling species distribution using maximum entropy

Maximum entropy is a machine learning algorithm that produces predictions of habitat suitability by comparing the conditional density of predictors at presence sites with the marginal density of predictors across the study area. The raw output of Maxent is a probability of habitat suitability [35,36]. To make model output more interpretable, Maxent converts the exponential values of its raw output estimate of habitat suitability into a logistic output that represents an estimate of probability of presence [37].

### Modeling species distribution using boosted regression trees

Boosted regression trees predict the value of a target variable based on the value of several input variables. Boosting uses a machine learning algorithm to produce a final prediction model that is an ensemble of weak prediction models, in this case, an ensemble of regression trees. The model is built in a stage wise fashion; a regression tree is fit to the original data, then the residuals of that model become the new data values, to which a second tree is fit, and so forth. By fitting each subsequent tree in the model to the residuals of the previous tree, the data become re-weighted in each iteration. Points that were misclassified by the previous model will now have more weight than values that were classified correctly. As a result, the subsequent tree will focus on fitting these misclassified points. The process is conditioned by the learning rate, which controls the contribution of each tree to the final model.

The final model can consist of thousands of trees, however, over-fit models will exaggerate minor fluctuations in the data, making them poor predictors. Boosted regression uses cross validation to minimize over-fitting by determining when adding additional trees no longer improves predictive performance, and selecting that optimum number of trees.

Predictive error is measured as the Bernoulli residual deviance between the predicted values of the model and the observed values of the test data. All BRT models are fitted in R using the ‘gbm’ and ‘dismo’ libraries [38-40].

### Target species and occurrence data

**Figure 1:**
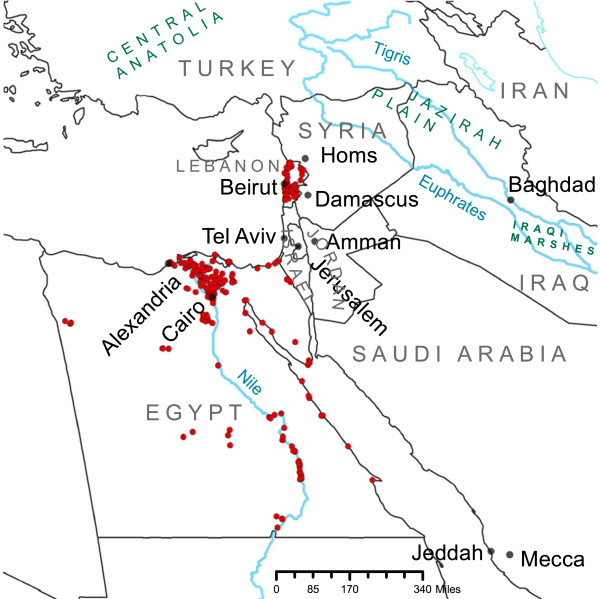
**Sampling locations of *Culex pipiens*.**

Our study area of interest (Figure 1) focuses on an approximately 5 million square kilometer region of the MENA that encompasses Egypt, Israel, Lebanon, Syria, Jordan as well as substantial portions of Turkey, Saudi Arabia, and Iraq.

The *Cx. pipiens* complex are the most widely distributed mosquito species in the world and two biotypes of *Cx. pipiens* L. are present in our study area, the anautogenous and autogenous biotypes. These mosquito biotypes breed in overlapping niches and readily hybridize in areas where they coexist [41]. In Egypt, both autogenous and anautogenous *Cx. pipiens* individuals were encountered in the progeny of autogenous or anautogenous female parents and more than 75% of field caught females produced mixed progenies [42].

*Cx. pipiens* is the most abundant mosquito in Lebanon, collected both indoors and outdoors. Active and abundant year round, it is anthropophilic, endophagic, and endophillic [21]. The species is highly behaviorally plastic, while *Cx. pipiens* in the Al Sharqyia governorate of Egypt’s Nile delta also displays endophagic and primarily anthropophiliic behavior [24], mosquitoes in the neighboring governorate have been observed to switch from indoor to outdoor feeding with seasonal shifts in temperature and wind speed [43]. Females will feed from a diverse range of hosts, including horses, cows, sheep, dogs, cats, humans and rats [23].

*Cx. pipiens* breeds in water with high organic content [41]. In Lebanon breeding sites for *Cx. pipiens* include containers which range in size from small cans filled with water to garden pools and irrigation ditches [21]. In the urban habitat of Cairo, the common breeding habitats for *Cx. pipiens* are cesspits, drainage canals, springs, cesspools, and irrigation ditches [22].

Training data from both Egypt and Lebanon were included to maximize sampling of the species’ environmental range, from the hot and hyperarid to cool and subhumid. This range of samples maximizes the combinations of environmental factors used in calibrating the models, increasing their predictive power [44,45].

Occurrence records for *Cx. pipiens* were obtained from previously unpublished vector surveys in Egypt and Lebanon (Figure 1). Adult samples from Lebanon were collected in the summer of 2009. Methods included CDC light traps with a dry ice attractant, and BG-sentinel traps with pheromone attractant. Traps were placed in outdoor and indoor habitats at 5 pm and collected the following morning. In addition to adult sampling, larval stages of mosquitoes were sampled in the summers of 2002 and 2003. Samples were collected by the classical dipping method using a white tray and/or a ladle from both artificial and natural breeding sites including swamps, ponds, riverbanks, irrigation tanks, and water storage tanks. Coordinates for sampling locations were obtained from Google Earth and GPS. Adult specimens were pinned and 4^th^ instar larval specimens were mounted on slides and identified morphologically using local and regional identification keys [46]. Occurrence records from Egypt (Gad, unpublished data) were collected using similar methods as part of a nation-wide survey.

### Environmental layers

We considered twenty-four environmental variables as potential predictors of *Cx. pipiens* habitat (Table 1). Temperature and precipitation were represented by 19 variables of the WorldClim dataset [47]. Quality of vegetative cover was described by the standard deviation, mean, and maximum values of the enhanced vegetation index (EVI), as derived from Moderate Resolution Image Spectroradiometer (MODIS) imagery from the Terra Satellite for 2001. The EVI provides a measure of photosynthetic activity or landscape greenness and hence captures vegetation features such as leaf area, canopy cover, sugar resources that may provide resting sites and alternate food sources for this vector species [48-50]. As available soil water limits primary productivity in the study region, mean and maximum EVI are generally correlated with annual rainfall, whereas the standard deviation of EVI provides a measure of vegetation seasonality, which is controlled by a variety of factors including temperature, day length, insolation, irrigation, and biotic factors [51]. An additional covariate related to potential vector breeding habitat was derived from topographic data, the topographical wetness index (TWI), which describes the tendency of water to collect in areas of topographic minima [52]. Because information regarding the distribution of hosts greatly improves models of mosquito distributions [53] and *Cx. pipiens* is adapted to breeding and feeding in human-altered landscapes, we also included human population density as a predictor using data from Landscan™ 2010. Environmental layers were gridded to a spatial resolution of approximately 1 km (926.63 m), a scale that captured as much environmental heterogeneity as possible within the limits of computer processing capability.

**Table 1:** Data sources of environmental predictors used in species distribution models

**Variable code**	**Data type**	**Date**	**Source res**	**Data source**
Bio1	Annual mean temperature	1960-1990	30 arc sec	WorldClim^1^
Bio2	Mean diurnal range	1960-1990	30 arc sec	WorldClim^1^
Bio3	Isothermality	1960-1990	30 arc sec	WorldClim^1^
Bio4	Temperature seasonality	1960-1990	30 arc sec	WorldClim^1^
Bio5	Maximum temperature of the warmest month	1960-1990	30 arc sec	WorldClim^1^
Bio6	Minimum temperature of the coldest month	1960-1990	30 arc sec	WorldClim^1^
Bio7	Temperature annual range	1960-1990	30 arc sec	WorldClim^1^
Bio8	Mean temperature of the wettest quarter	1960-1990	30 arc sec	WorldClim^1^
Bio9	Mean temperature of the driest quarter	1960-1990	30 arc sec	WorldClim^1^
Bio10	Mean temperature of the warmest quarter	1960-1990	30 arc sec	WorldClim^1^
Bio11	Mean temperature of the coldest quarter	1960-1990	30 arc sec	WorldClim^1^
Bio12	Annual precipitation	1960-1990	30 arc sec	WorldClim^1^
Bio13	Precipitation of the wettest month	1960-1990	30 arc sec	WorldClim^1^
Bio14	Precipitation of the driest month	1960-1990	30 arc sec	WorldClim^1^
Bio15	Precipitation seasonality	1960-1990	30 arc sec	WorldClim^1^
Bio16	Precipitation of the wettest quarter	1960-1990	30 arc sec	WorldClim^1^
Bio17	Precipitation of the driest quarter	1960-1990	30 arc sec	WorldClim^1^
Bio18	Precipitation of the warmest quarter	1960-1990	30 arc sec	WorldClim^1^
Bio19	Precipitation of the coldest quarter	1960-1990	30 arc sec	WorldClim^1^
EVIMAX	Maximum EVI	2001	250 m	MODIS^2^
EVIMEAN	Average annual EVI	2001	250 m	MODIS^2^
EVISD	Standard deviation of EVI	2001	250 m	MODIS^2^
Population	Population count	2010	30 arc sec	Landscan^3^
TWI	Topographical wetness index	2000	90 m	GLSDEM^4^

^1^WorldClim Global Climate database v1.4, available at :http://www.worldclim.org/ {accessed 28/8/2013}.

^2^Moderate Resolution Imaging Spectrometer (MODIS), available at: https://lpdaac.usgs.gov/ {accessed 28/8/2013}.

^3^LandScan (2010)™ High Resolution global Population Data Set copyrighted by UT-Battelle, LLC, operator of Oak Ridge National Laboratory under Contract No. DE-AC05-00OR22725 with the United States Department of Energy.

^4^Global Land Survey Digital Elevation Model (GLSDEM), available at: http://www.glcf.umd.edu/data/glsdem/ {accessed 28/8/2013} (Input elevation data set to GLSDEM for study area is Shuttle Radar Topography Mission (SRTM)).

Data sources of environmental predictors used in construction of species distribution models. All layers were gridded to 926.63 m spatial resolution and projected into the MODIS sinusoidal projection.

### Data processing

Presence Data: All point locations recorded as positive for *Cx. pipiens* from Egypt (n = 239) and Lebanon (n = 83) surveys were included as presence locations in all models (Figure 1).

Background Data: Both boosted regression and maximum entropy methods predict areas with a high probability of presence of conditions suitable for the target species by comparing the values of environmental predictors at presence locations to the values of the same predictors at a set of random “background” locations, which represent the range of available environmental values [54]. Because our data, like many presence-only data sets [55,56], showed a strong bias in sampling effort, we selected background points with the same geographical bias as the occurrence data (Additional file 1).

An independent data set of 79 *Cx. pipiens* locations, 23 from Israel and 56 from Egypt [57], was used to assess accuracy of model predictions (Additional file 2: Figure S1). These data were used in all Maxent test data calculations.

### Assessing the contribution of human population density to *Cx. pipiens* distribution models

Human population density has the potential to be a strong predictor of *Cx. pipiens* habitat [53,58]. However, because the survey locations of the original studies were all in close proximity to populated areas, population density is also a strong predictor of sampling effort. To control for this we ran the entire modeling process twice; once on the complete set of environmental variables (N = 24), and once using all environmental variables except population density (N = 23). Including population density allows us to examine the relative influence of an important socioeconomic environmental factor, while excluding human populations allows us to examine the bio-physical environmental factors on their own.

To increase the interpretability of the models both predictor sets were reduced using the ‘gbm.simplify’ function, which creates BRT models with every possible combination of the initial predictors, and based on minimizing predictive error, ranks predictors from most to least influential, and identifies the optimal predictor set [59].

### Creating species distribution models

BRT models using the reduced predictor sets were fitted using a learning rate of 0.05, a tree complexity of 5, a bag fraction of 0.75, 10-fold cross validation, and a Bernoulli loss function. All other parameters were left at default settings. Predictions in geographic space were made in R using the “raster” package [60].

Maxent models using the reduced predictor sets were fit using Maxent v 3.3.3 k [61]. Spatial predictions of the model were "clamped", a Maxent feature which reduces the value of the predictions when extrapolating into areas of parameter space that exceeded the range of values present in the training data. The final model was the average logistic output of 10 repetitions of the modeling process. Additional specifics on program specifications can be found in Additional file 1.

### Evaluating and comparing model predictions

Maxent models were evaluated using the area under the receiver operating characteristic curve (AUC), the corrected Aikaike information criterion (AICc), and the omission rate. BRT models were evaluated using AUC, point biserial correlation (COR), and the Bernoulli deviance. Because the two techniques use different test points for the intrinsic measures of model performance, the point biserial correlation was also calculated for all models at a common set of 158 points made of the 79 test presence locations and 79 random background points (Additional file 2: Figure S1). Pearson’s correlation coefficient was used to measure how closely predictions agreed between the two methods. Agreement was compared in the “training region”, using the common 158 points, as well as an “expanded region” of 158 points drawn from the entire model extent (Additional file 2: Figure S1).

## Results

### *Cx. pipiens* habitat suitability

**Figure 2:**
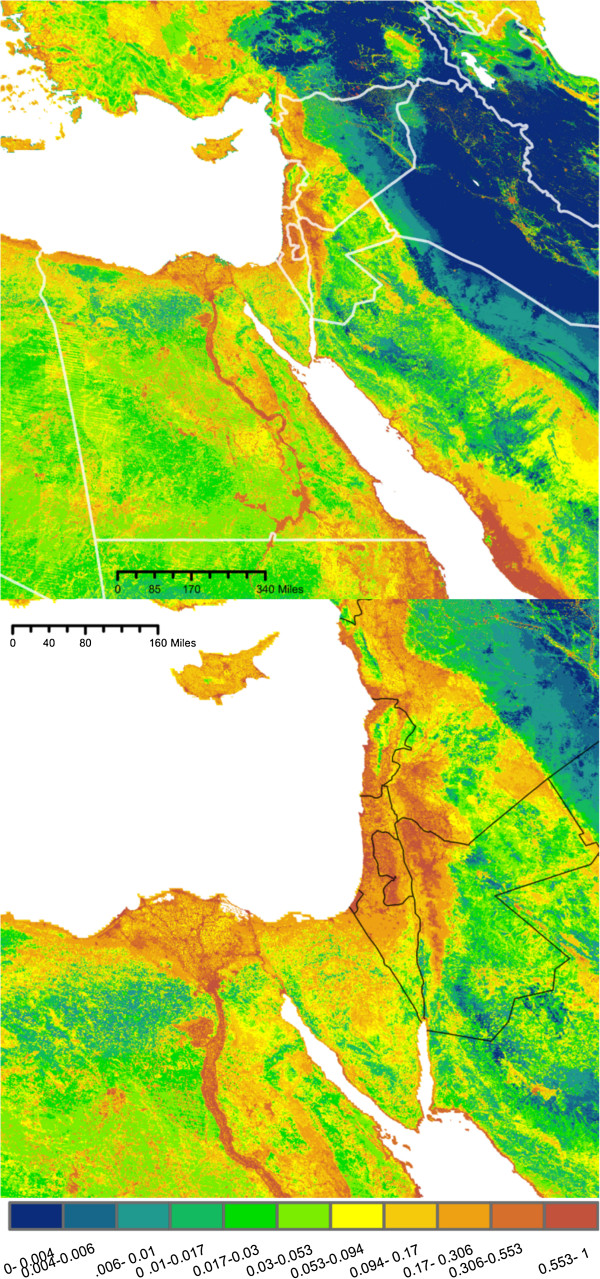
**Maxent Pop. Probability of *Culex pipiens *presence based on a species distribution model generated using maximum entropy and a set of environmental predictors (N = 9) including human population density.** Habitat suitability has been converted to probability of presence, assuming a prevalence of 0.5. Bottom: Enlarged to show population centers.

**Figure 3:**
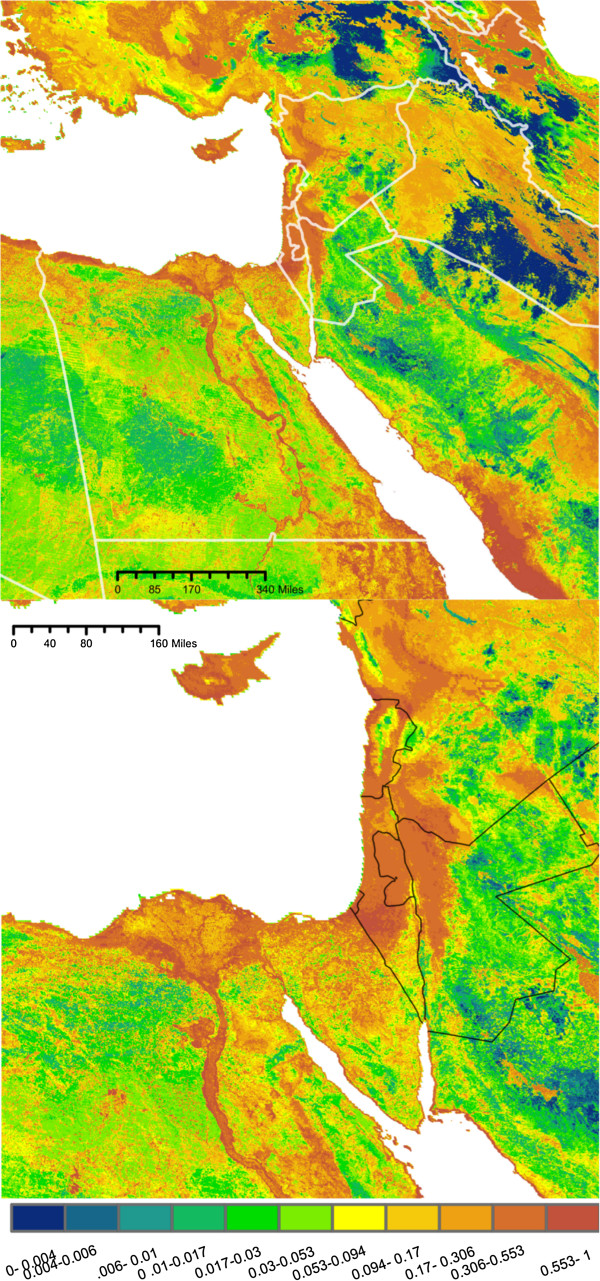
**Maxent No Pop. Probability of *Culex pipiens *presence based on a species distribution model generated using maximum entropy and a set of environmental predictors (N = 14) that do not include human population density.** Habitat suitability has been converted to probability of presence, assuming a prevalence of 0.5. Bottom: Enlarged to show population centers.

**Figure 4:**
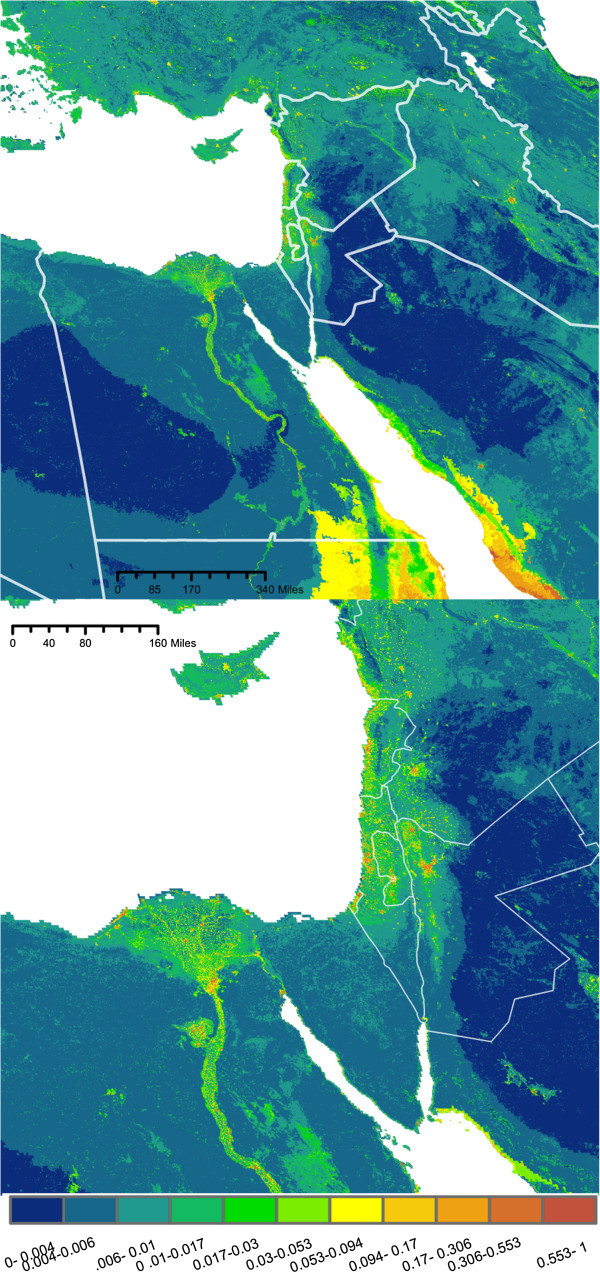
**BRT Pop. Probability of *Culex pipiens *presence based on a species distribution model generated using boosted regression trees and a set of environmental predictors (N = 9) that includes include human population density.** Bottom: Enlarged to show population centers.

**Figure 5:**
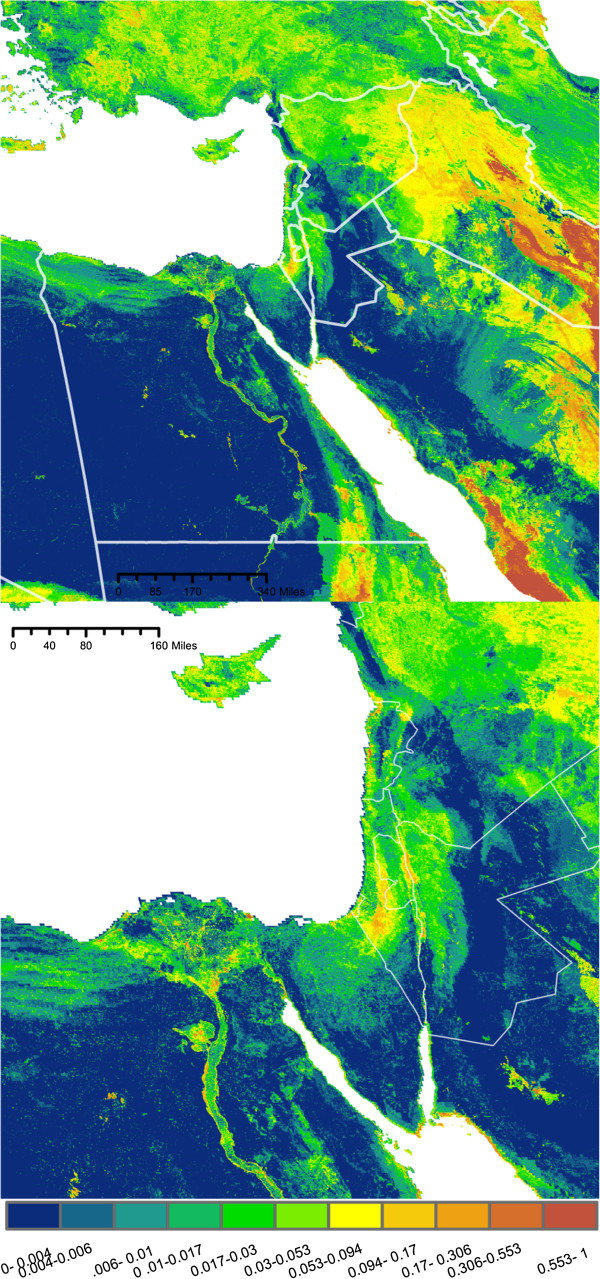
**BRT No Pop: Probability of *Culex pipiens *presence based on a species distribution model generated using boosted regression trees and a set of environmental predictors (N = 14) that does not include human population density.** Bottom: Enlarged to show population centers.

The predicted maps of suitable *Cx. pipiens* habitat are presented in Figures 2, 3, 4 and 5. Overall, maximum entropy models predicted a broader distribution of suitable habitat than boosted regression methods, which produced much more conservative predictions extent of suitable habitat and a lower probability of presence. However, relative suitability of habitats was consistent between models: areas of highest suitability identified by BRT were also the areas of highest suitability selected by maximum entropy.

All four models agree in predicting a moderate to high probability of *Cx. pipiens* presence in several distinct habitats: along the banks of the Nile and throughout the Nile Delta, throughout the coastal plains of Israel, Lebanon, and Syria, as well as the valleys of east Lebanon and along Israel’s Jordanian border (Figures 2, 3, 4, and 5). In models built using human population data (Figures 2 and 4), habitats with the highest probability of presence were found near large population centers. Both BRTPop and MaxentPop models also indicate moderate probability of presence along the banks of the Tigris and Euphrates rivers. Models built without population data (Figures 3 and 5) predicted greater probability of presence in the semi-arid Central Anatolia region of Turkey, the Jazirah plain in North East Syria and Northern Iraq, and the marshes of southern Iraq than the population-inclusive models. However, much of Iraq and Eastern Turkey occupy a region of environmental space that falls outside the range of the training data (Additional file 2: Figures S3 and S4), so predictions in these areas should be interpreted cautiously.

### Model accuracy

Evaluation metrics for Maxent and BRT models are presented in Tables 2 and 3. Among Maxent models, both sets of predictors performed well, with AUC values significantly greater than the null model. The test AUC values for both predictor sets were smaller than the training AUC values, which indicates slight over-fitting of the models. This difference was smaller for the model created without population data. The “NoPop” model also had a higher test AUC value, greater entropy, and a smaller omission rate than the model that included population. The population-inclusive model had a lower AICc than the “NoPop” model.

**Table 2:** Evaluation parameters for species distribution models built using human population data

**Model**	**Parameters in final model**	**Observed training AUC**	**Observed test AUC**	**Null model training AUC**	**Null model test AUC**	**Prevalence**	**Entropy**	**AICc**	**Omission rate**^**3 **^**N = 79**	**Test COR (N = 158)**	**Test deviance (N = 158)**
Maxent Pop^1^	9	0.938	0.872	0.827 ± 0.01	0.450 +/- 0.03	0.151	8.043	8383	0.206	0.470	197.9
Maxent NoPop^2^	14	0.916	0.879	0.848 ± 0.01	0.476 +/- 0.04	0.185	8.252	8916	0.194	0.534	178.3

^1^Data sources used for model development: Bioclim + Vegetation + Population (N = 24).

^2^Data sources used for model development: Bioclim + Vegetation (N = 23).

^3^Using equal test sensitivity and specificity threshold. Maxent Pop threshold =0.22, Maxent No Pop threshold =0.34.

Evaluation parameters for *Culex pipiens* distribution models generated using a set of environmental predictors including human population density (“Maxent Pop”) and a set excluding human population density (“Maxent No Pop”). Models were built using 322 presence points. “Null Model” parameters represent the average value of models built from one hundred 322-point dummy data sets. “Test AUC” and “Omission Rate” are calculated from an independent data set of 79 presence points, not used in training the models. “Test COR” and “Test Deviance” are calculated from a data set that includes the 79 test data points and 79 background points. “Test COR” = Pearson correlation coefficient.

**Table 3:** Evaluation parameters for species distribution models excluding human population data

**Model**	**Parameters in final model**	**#trees**	**Training AUC**	**CV AUC**	**Training COR**	**CV COR**	**CV deviance**	**Test COR (N = 158)**	**Test deviance (N = 158)**
BRT Pop^1^	9	1550	0.945	0.889	0.663	0.431	0.198	0.413	453.3
BRT No Pop^2^	14	3050	0.982	0.864	0.745	0.361	0.212	0.470	395.8

^1^Data sources used for model development: Bioclim + Vegetation + Population (N = 24).

^2^Data sources used for model development: Bioclim + Vegetation (N = 23).

Evaluation parameters for *Culex pipiens* distribution models generated using boosted regression trees from a set of environmental predictors including human population density (“BRT Pop”) and a set excluding human population density (“BRT No Pop”). Models were built using 322 presence points, and evaluated using 10 fold cross validation. “CV AUC” and “CV Deviance” are the area under the curve (AUC) and Bernoulli deviance evaluated at the withheld dataset. “Test COR” and “Test Deviance” are calculated from the predicted model values at 79 independent occurrence points not used in training the mode, and 79 background points from the same region. “Test COR” = Pearson correlation coefficient.

The boosted regression models also performed well, and also displayed a degree of over-fitting similar to the Maxent models. Among the BRT models, population-inclusive models performed slightly better than the “NoPop” models on test data- with higher cross-validated AUC values, higher cross-validated COR values, and lower cross validated deviance scores.

When evaluated over a standard set of points, the Maxent “NoPop” model had the strongest correlation (COR = 0.534) between model predictions and presence/pseudo-absence, the BRT NoPop and Maxent population-inclusive model performed equally well, and the BRT population-inclusive model had the weakest correlation (COR = 0.413) of the four models evaluated.

### Model agreement

**Figure 6:**
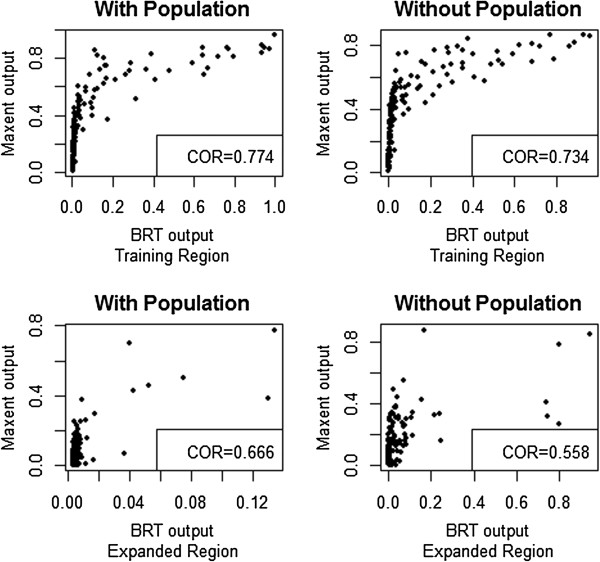
**Correlation between predictions of *Culex pipiens *distribution models created using boosted regression trees (BRT) and maximum entropy (Maxent) algorithms.** “Training region” consists of 158 points taken from within a similar geographical area to the samples used building the model, 79 background points and 78 independent occurrence points. “Expanded Region” measures model agreement throughout the entire modeled extent, and compares values at 158 random points taken with equal probability from the entire model extent. Left panels describe models built using human population density as a parameter (N = 9). Panels on the right describe models excluding human population density (N = 14). COR = Pearson correlation coefficient.

When evaluated over the training region, the predictions of Maxent and BRT models were very strongly correlated, with models including population data more closely correlated than models which excluded it (Figure 6). When evaluated over the entire modeled region, the correlation between models was weaker.

### Contribution of environmental predictors to *Cx. pipiens* distribution models

Parameter reduction of the full predictor set (n = 24) resulted in a simplified set of 9 significant predictors. (Table 4). Parameter reduction of the full predictor set excluding human population density (n = 23) resulted in a simplified set of 14 significant predictors (Table 5).

**Table 4:** Contribution of environmental parameters to SDMs including human population data

**Parameter**	**BRT variable importance**	**Maxent variable importance**	**Maxent permutation importance**	**BRT rank**	**Maxent rank**
Population	34.9	60.6	52.8	1	1
EVIMEAN	11.1	7.5	0.8	2	3
Bio8	9.1	6.1	0.5	3	5
EVIMAX	8.7	3.5	1.0	4	6
Bio12	8.4	6.2	3.4	5	4
EVISD	8.4	11.9	39.1	6	2
Bio4	7.1	1.2	1.3	7	8
Bio6	6.7	2.4	0.0	8	7
Bio7	5.6	0.7	1.0	9	9

Contribution of environmental parameters (Table 1) to species distribution models for *Culex pipiens* generated using boosted regression trees (BRT) and maximum entropy (Maxent) methods.

**Table 5:** Contribution of environmental parameters to SDMs excluding human population data

**Parameter**	**BRT variable importance**	**Maxent variable importance**	**Maxent permutation importance**	**BRT rank**	**Maxent rank**
EVISD	16.3	44.7	73.6	1	1
EVIMEAN	14.7	12.6	3.3	2	2
EVIMAX	9.7	7.9	11.3	3	4
TWI*	9.4	2.9	1.9	4	9
Bio4	8.2	1.8	5.5	5	10
Bio10*	5.7	3.1	0.1	6	8
Bio15*	5.0	3.3	1.8	7	7
Bio12	5.0	4.6	0.1	8	5
Bio8	4.9	11.7	0.2	9	3
Bio7	4.7	0.7	0.1	10	13
Bio2*	4.3	0.4	0.5	11	14
Bio6	4.3	3.6	0.5	12	6
Bio5*	4.1	1.6	0.0	13	11
Bio9*	3.7	1.0	1.0	14	12

^*^Environmental features not utilized in population-inclusive models (Table 4).

Contribution of environmental parameters (Table 1) to species distribution models for *Culex pipiens* generated using boosted regression trees (“BRT”) and maximum entropy (“Maxent”) methods.

Both modeling techniques selected similar variables as the most important environmental factors driving species distribution. Population was the largest single contributor to model predictions, ranked as most influential under both BRT (34.9%) and Maxent (60.6%).

In models created without human population density, the variable with the single largest contribution was the standard deviation of the enhanced vegetation index (EVI). Mean EVI was also highly ranked in all models and all data sets.

## Discussion

### Model agreement and predictions

Our models showed strong agreement between boosted regression and maximum entropy methods in selection of habitat with the highest probability of occurrence of *Cx. pipiens*. High AUC values for all four models indicated that occupancy sites were highly likely to be assigned a higher probability of presence than background sites regardless of method. The strong correlation between BRT and Maxent outputs in areas with a high proportion of known *Cx. pipiens* habitat indicates that the models strongly agree on areas of potentially high risk. These areas included populated areas within the Nile delta, the valleys of Israel, Lebanon, and Jordan, and southwestern Saudi Arabia.

Our model predictions are supported by agreement between predicted areas of highest probability of *Cx. pipiens* occurrence, and recent positive vector surveys, or outbreaks of vector-associated diseases. The regions of central Israel, predicted by our models to be areas of high probability of *Cx. pipiens* presence, corresponded to the areas of highest incidence of West Nile virus during the 2000 outbreak [8]. Likewise, the region of highest probability of occupancy in Saudi Arabia corresponds with recent collections of *Cx. pipiens*[62-64] and the incidence of infection during the 2000 Rift Valley Fever virus outbreak [65]. The distribution of suitable *Cx. pipiens* habitat in Saudi Arabia is of special interest because of the high potential for repeated import of diseases into the country via large numbers of pilgrims travelling through for the annual Hajj [66]. The city of Jeddah has twice been hit by epidemics of dengue directly following the Hajj [67]. Our results indicate that this same area is a suitable habitat for supporting *Cx. pipiens*, and potentially capable of transmitting West Nile virus, should it be re-introduced.

Agreement between model predictions was less correlated when evaluated over the full region, including areas where models were required to extrapolate. This is a result of the different behavior of each algorithm in extreme environments [68]. Maxent, when clamped, extrapolates in a horizontal line from the fit at the most extreme value in the training data. BRT, which does not use clamping, extrapolates at a constant value from the last known site. This difference in extrapolation can also be seen in the spatial predictions of the population-exclusive models when predicting into the most dissimilar areas of the modeling region. BRT assigns a higher relative probability of occupancy to habitat in northern Iraq and southern Syria, classifying the area with a probability of occupancy similar to that of the river valley along the Israel-Jordan border. The Maxent model, which constrains its predictions more severely, predicts a much more moderate probability of occupancy. The same relative over-estimation of the BRT algorithm is not as apparent in the population-inclusive model, because the environmental predictor that most exceeds its training values in this region is temperature seasonality, which was not as influential a predictor in the population-inclusive models as it was in the population-exclusive models. The effect of clamping can still be seen in the population-inclusive Maxent model. An advantage of using two different modeling methods is our ability to detect regions of parameter space where choice of the underlying modeling algorithm has the greatest influence on strength of predictions. Results in these areas need to be interpreted more cautiously than areas where both models are in agreement.

Presence-only modeling methods, such as those used in this study, make the assumption that a target species is equally detectable across all habitats [35]. However, if sampling detection probability varies with one or more environmental predictors, our model will not distinguish between habitat with a higher probability of occupancy and a habitat with greater detectability. Absolute values of the predictions should be interpreted with caution with this limitation in mind. Predictions should also be evaluated with the consideration that presence-only models treat densely and sparsely occupied sites the same, as the input data are binary.

In order to efficiently predict species distributions across the study area, we were required to coarsen the resolution of environmental predictors to 1 km. It is important to keep in mind that this may be a larger than optimal scale for examining the relationship between vectors and certain predictors with very fine scale heterogeneity [25,69].

### Relationship between *Cx. pipiens* and environmental predictors

Our study is unique in its aim of identifying the underlying environmental predictors driving *Cx. pipiens* distribution across a climate gradient that encompasses hyper arid to sub humid habitats. The novel aspects of our findings are the importance of human population density and seasonality of vegetation indices as powerful predictors of *Cx. pipiens* occurrence.

Human population density was identified by both modeling methods as the highest contributing predictor to habitat suitability. Interpreting this result is complex, because areas of dense human habitation may be favorable to mosquitoes for several reasons. Humans are a host species, their dwellings also provide a sheltered resting area, and in rural areas human activities such as irrigation and agriculture may also provide favorable breeding habitat and non-human hosts. Breeding conditions for mosquitoes are also favorable in slums, where high human population density is combined with poor infrastructure and inadequate services [70]. Characterization of mosquito breeding habitat in urban Cairo found that 93.5% of breeding sites were found in slum areas, characterized by incomplete construction, disorganized roads, and crowded, dense conditions [71]. Socioeconomic conditions were also significantly related to *Cx. pipiens* breeding sites in Washington DC, although in the northern temperate region the relationship was reversed, breeding sites were positively associated with presence of functional containers, like flower pots and garbage pails, which were found primarily in areas of higher socioeconomic status [27].

Our results indicate that quality and seasonality of vegetation is a powerful predictor of *Cx. pipiens* in arid and semi-arid habitats. The relationship between *Cx. pipiens* occurrence and vegetation quality was strongly positively associated with the maximum value of the enhanced vegetation index (EVI), and negatively associated with the mean and standard deviation (seasonality) of the EVI. This suggests that in our study region, high quality *Cx. pipiens* habitat consists of areas of high primary productivity, which maintain that quality of habitat with very little change throughout the year. In rural areas of Egypt, year round irrigation maintains precisely this kind of habitat. Vegetation indices are not always the most informative predictors in the region, in a study examining another *Cx. pipiens* vectored infection, filariasis, the Normalized Difference Vegetation Index did not distinguish between Egyptian villages at high and low risk of infection [72]. The positive relationship between *Cx. pipiens* and stable areas of high primary productivity suggested by our results is the opposite of the relationship seen in the forested northeastern United States, where the most accurate model of *Cx. pipiens* abundance was negatively correlated with forest cover and did not include the vegetative index at all [25].

Topographic wetness index has been a significant predictor of anopheline abundance and malaria risk among households in Thailand [73] and Kenya [74,75]. Despite this, in our models it was a significant predictor only in the BRT "NoPop" model. It is possible that in arid climates with low humidity and little rainfall, the potential for pooling water, as predicted by TWI, does not translate into pools of water with enough frequency to make TWI a stronger indicator of vector breeding habitat. It is also possible that, even if TWI does accurately predict the distribution of pools of clear groundwater in the region, those pools of clear groundwater may not be the breeding habitat most favored by *Cx. pipiens*. In habitats where mosquitos chose to oviposit more frequently in artificial or natural containers, rather than ground pools, this will not be reflected in the TWI. It is also notable that none of our models found rainfall to be a very powerful predictor. In this context, the vegetation indices may provide better information on available soil moisture than hydrology or rainfall parameters.

## Conclusions

Our study provides insight into the drivers of *Cx. pipiens* distribution in an understudied region at growing risk from the re-emergence of several arboviruses. The most dominant predictors in our species distribution model, human population density and the seasonality of the enhanced vegetation index, are also both environmental factors with potential correlations with urbanization and agricultural practices. By understanding the relationship between these predictors and disease vector distributions, we gain a better understanding of how changes in land use or shifting patterns of human settlement might influence disease transmission. Given the resurgence of vector borne diseases like West Nile and Rift Valley Fever [10] understanding how choices may influence disease ecology through agriculture, urbanization, and population growth, is a topic of vital importance.

## Abbreviations

SDM: Species distribution model; BRT: Boosted regression tree; MENA: Middle East and North Africa; MODIS: Moderate Resolution Image Spectroradiometer; EVI: Enhanced Vegetative Index; TWI: Topographic Wetness Index; VBD: Vector borne diseases.

## Competing interests

The authors declare that they have no competing interests.

## Authors’ contributions

AC designed the study, developed the species distribution models, performed the statistical analyses and drafted the manuscript. DO conceived of the study, participated in its design, acquired remotely sensed data and extracted summary data from MODIS imagery, and helped to draft the manuscript. NH conducted vector surveys (Lebanon) and helped draft the manuscript. AG conducted vector surveys (Egypt). AH organized vector data (Egypt) and provided significant revisions to the manuscript. JB conceived of the study, participated in its design and coordination, and helped draft the manuscript. All authors read and approved the final manuscript.

## Supplementary Material

Additional file 1 Additional parameters used for generation and evaluation of species distribution models. Click here for file

Additional file 2 Supplemental maps of background points and additional Maxent output. Click here for file

## References

[B1] Cohen B Urban growth in developing countries: a review of current trends and a caution regarding existing forecasts World Dev 2004 32 23 51 10.1016/j.worlddev.2003.04.008

[B2] Hotez PJ Savioli L Fenwick A Neglected tropical diseases of the Middle East and North Africa: review of their prevalence, distribution, and opportunities for control PLoS Negl Trop Dis 2012 6 e1475 10.1371/journal.pntd.0001475 22389729 PMC3289601

[B3] Roudi FN Population Trends and Challenges in the Middle East and North Africa 2001 Washington, DC: Population Reference Bureau

[B4] Reiter P Climate change and mosquito-borne disease Environ Health Persp 2001 109 Suppl 1 141 161 10.1289/ehp.01109s1141 PMC1240549 11250812

[B5] Meegan JM The Rift Valley fever epizootic in Egypt 1977-78. 1. Description of the epizzotic and virological studies Trans R Soc Trop Med Hyg 1979 73 618 623 10.1016/0035-9203(79)90004-X 538803

[B6] Hoogstraal H Meegan JM Khalil GM Adham FK The Rift Valley fever epizootic in Egypt 1977-78. 2. Ecological and entomological studies Trans R Soc Trop Med Hyg 1979 73 624 629 10.1016/0035-9203(79)90005-1 44038

[B7] Shoemaker T Boulianne C Vincent MJ Pezzanite L Al-Qahtani MM Al-Mazrou Y Khan AS Rollin PE Swanepoel R Ksiazek TG Nichol ST Genetic analysis of viruses associated with emergence of Rift Valley fever in Saudi Arabia and Yemen, 2000-01 Emerg Infect Dis 2002 8 1415 1420 10.3201/eid0812.020195 12498657 PMC2738516

[B8] Weinberger M Pitlik SD Gandacu D Lang R Nassar F David DB Rubinstein E Izthaki A Mishal J Kitzes R West Nile fever outbreak, Israel, 2000: epidemiologic aspects Emerg Infect Dis 2001 7 686 10.3201/eid0704.017416 11585533 PMC2631774

[B9] Paz S Semenza JC Environmental drivers of West Nile fever epidemiology in Europe and Western Asia–a review IJERPH 2013 10 3543 3562 10.3390/ijerph10083543 23939389 PMC3774453

[B10] Harrus S Baneth G Drivers for the emergence and re-emergence of vector-borne protozoal and bacterial diseases Int J Parasitol 2005 35 1309 1318 10.1016/j.ijpara.2005.06.005 16126213

[B11] Cosner C Beier JC Cantrell RS Impoinvil D Kapitanski L Potts MD Troyo A Ruan S The effects of human movement on the persistence of vector-borne diseases J Theor Biol 2009 258 550 560 10.1016/j.jtbi.2009.02.016 19265711 PMC2684576

[B12] Apperson CS Hassan HK Harrison BA Savage HM Aspen SE Farajollahi A Crans W Daniels TJ Falco RC Benedict M Anderson M McMillen L Unnasch TR Host feeding patterns of established and potential mosquito vectors of West Nile virus in the eastern United States Vector-Borne Zoonot Dis 2004 4 71 82 10.1089/153036604773083013 PMC2581457 15018775

[B13] Taylor B Work TH Hurlbut HS Rizk F A study of the ecology of West Nile virus in Egypt Am J Trop Med Hyg 1956 5 579 620 13354882 10.4269/ajtmh.1956.5.579

[B14] Turell MJ Presley SM Gad AM Cope SE Dohm DJ Morrill JC Arthur RR Vector competence of Egyptian mosquitoes for Rift Valley fever virus Am J Trop Med Hyg 1996 54 136 139 8619436 10.4269/ajtmh.1996.54.136

[B15] Kilpatrick AM Kramer LD Campbell SR Alleyne EO Dobson AP Daszak P West Nile virus risk assessment and the bridge vector paradigm Emerg Infect Dis 2005 11 425 429 10.3201/eid1103.040364 15757558 PMC3298247

[B16] Amraoui F Krida G Bouattour A Rhim A Daaboub J Harrat Z Boubidi S-C Tijane M Sarih M Failloux A-B *Culex pipiens*, an experimental efficient vector of West Nile and Rift Valley fever viruses in the Maghreb region PLoS One 2012 7 e36757 10.1371/journal.pone.0036757 22693557 PMC3365064

[B17] Tran A Ippoliti C Balenghien T Conte A Gely M Calistri P Goffredo M Baldet T Chevalier V A geographical information system-based multicriteria evaluation to map areas at risk for Rift Valley fever vector-borne transmission in Italy Transbound Emerg Dis 2013 60 14 23 24589097 10.1111/tbed.12156

[B18] Kalluri S Gilruth P Rogers D Szczur M Surveillance of arthropod vector-borne infectious diseases using remote sensing techniques: a review PLoS Pathog 2007 3 e116 10.1371/journal.ppat.0030116 PMC2042005 17967056

[B19] Amr ZS Al-Khalili Y Arbaji A Larval mosquitoes collected from northern Jordan and the Jordan Valley J Am Mosquito Contr 1997 13 375 378 9474565

[B20] Al-Khalili YH Katbeh-Bader A Mohsen ZH Siphon index of *Culex pipiens* larvae collected from different biogeographical provinces in Jordan Zool Middle East 1999 17 71 76 10.1080/09397140.1999.10637770

[B21] Knio KM Markarian N Kassis A Nuwayri-Salti N A two-year survey on mosquitoes of Lebanon Parasite 2005 12 229 235 10.1051/parasite/2005123229 16218210

[B22] Ammar SE Kenawy MA Abdel-Rahman HA Gad AM Hamed AF Ecology of the mosquito larvae in urban environments of Cairo Governorate, Egypt J Egypt Soc Parasitol 2012 42 191 202 22662608 10.12816/0006307

[B23] Bhagat IM Host-feeding patterns of *Culex pipiens* (Diptera-Culicidae) in El-Abtal village, Ismailia Governorate, Egypt Egypt J Biol 2004 2 139 143

[B24] Gad AM Riad IB Farid HA Host-feeding patterns of *Culex pipiens* and *Cx. antennatus* (Diptera: Culicidae) from a village in Sharqiya Governorate, Egypt J Med Entomol 1995 32 573 577 7473609 10.1093/jmedent/32.5.573

[B25] Diuk-Wasser M Brown H Andreadis T Fish D Modeling the spatial distribution of mosquito vectors for West Nile virus in Connecticut, USA Vector-Borne Zoonot 2006 6 283 295 10.1089/vbz.2006.6.283 16989568

[B26] Mweya CN Kimera SI Kija JB Mboera LEG Predicting distribution of *Aedes aegypti* and *Culex pipiens* complex, potential vectors of Rift Valley fever virus in relation to disease epidemics in East Africa Infect Ecol Epidemiol 2013 3 109 10.3402/iee.v3i0.21748 PMC3797365 24137533

[B27] Dowling Z Ladeau SL Armbruster P Biehler D Leisnham PT Socioeconomic status affects mosquito (Diptera: Culicidae) larval habitat type availability and infestation level J Med Entomol 2013 50 764 772 10.1603/ME12250 23926774

[B28] Farid HA Morsy ZS Hassan AN Hammad RE Faris R Kandil AM Ahmed ES Weil GJ The impact of environmental and entomological factors on intervillage filarial focality in the Nile Delta J Egypt Soc Parasitol 2000 30 469 485 10946509

[B29] Gatton ML Kay BH Ryan PA Environmental predictors of Ross River virus disease outbreaks in Queensland, Australia Am J Trop Med Hygiene 2005 72 792 799 15964965

[B30] Morin CW Comrie AC Regional and seasonal response of a West Nile virus vector to climate change Proc Natl Acad Sci USA 2013 110 15620 15625 10.1073/pnas.1307135110 24019459 PMC3785720

[B31] Pearson RG Raxworthy CJ Nakamura M Townsend Peterson A Predicting species distributions from small numbers of occurrence records: a test case using cryptic geckos in Madagascar J Biogeogr 2007 34 102 117

[B32] Kumar S Stohlgren TJ Maxent modeling for predicting suitable habitat for threatened and endangered tree Canacomyrica monticola in New Caledonia J Ecol Nat Environ 2009 1 94 98

[B33] Khatchikian C Sangermano F Kendell D Livdahl T Evaluation of species distribution model algorithms for fine-scale container-breeding mosquito risk prediction Med Vet Entomol 2010 25 268 275 21198711 10.1111/j.1365-2915.2010.00935.x PMC3135728

[B34] Elith J Phillips SJ Hastie T Dudík M Chee YE Yates CJ A statistical explanation of MaxEnt for ecologists Divers Distrib 2010 17 43 57

[B35] Yackulic CB Chandler R Zipkin EF Royle JA Nichols JD Campbell Grant EH Veran S Presence-only modelling using MAXENT: when can we trust the inferences? Methods Ecol Evol 2012 4 236 243

[B36] Fitzpatrick MC Gotelli NJ Ellison AM MaxEnt versus MaxLike: empirical comparisons with ant species distributions Ecosphere 2013 4 1 15

[B37] Phillips SJ Dudík M Modeling of species distributions with Maxent: new extensions and a comprehensive evaluation Ecography 2008 31 161 175 10.1111/j.0906-7590.2008.5203.x

[B38] R Development Core Team R: A language and environment for statistical computing 2013

[B39] Hijmans RJ Phillips S Leathwick J Elith J Dismo: Species Distribution Modeling 2013 R package version 0.9-1

[B40] Ridgeway G Gbm: Generalized Boosted Regression Models 2013 R package version 2.1

[B41] Farajollahi A Fonseca DM Kramer LD Kilpatrick AM “Bird biting” mosquitoes and human disease: a review of *Culex pipiens* complex mosquitoes in epidemiology Infect Genet Evol 2011 11 1577 1585 10.1016/j.meegid.2011.08.013 21875691 PMC3190018

[B42] Gad AM Abdel Kader M Farid HA Hassan AN Absence of mating barriers between autogenous and anautogenous *Culex pipiens* L. in Egypt J Egypt Soc Parasitol 1995 25 63 71 7602173

[B43] Gad AM Feinsod FM Soliman BA el Said S Survival estimates for adult *Culex pipiens* in the Nile Delta Acta Trop 1989 46 173 179 10.1016/0001-706X(89)90034-X 2566270

[B44] Thuiller W Brotons L Araújo MB Lavorel S Effects of restricting environmental range of data to project current and future species distributions Ecography 2004 27 165 172 10.1111/j.0906-7590.2004.03673.x

[B45] Pearson RG Dawson TP Predicting the impacts of climate change on the distribution of species: are bioclimate envelope models useful? Global Ecol Biogeogr 2003 12 361 371 10.1046/j.1466-822X.2003.00042.x

[B46] Harbach RE The mosquitoes of the subgenus Culex in southwestern Asia and Egypt (Diptera: Culicidae) Contrib Am Entomol Inst 1988 24 1 140

[B47] Hijmans RJ Cameron SE Parra JL Jones PG Jarvis A Very high resolution interpolated climate surfaces for global land areas Int J Climatol 2005 25 1965 1978 10.1002/joc.1276

[B48] Gu W Müller G Schlein Y Novak RJ Beier JC Natural plant sugar sources of Anopheles mosquitoes strongly impact malaria transmission potential PLoS One 2011 6 e15996 10.1371/journal.pone.0015996 21283732 PMC3024498

[B49] Andersson IH Jaenson TGT Nectar feeding by mosquitoes in Sweden, with special reference to *Culex pipiens* and *Cx torrentium* Med Vet Entomol 1987 1 59 64 10.1111/j.1365-2915.1987.tb00323.x 2979521

[B50] Foster WA Mosquito sugar feeding and reproductive energetics Annu Rev Entomol 1995 40 443 474 10.1146/annurev.en.40.010195.002303 7810991

[B51] Richardson AD Keenan TF Migliavacca M Ryu Y Sonnentag O Toomey M Climate change, phenology, and phenological control of vegetation feedbacks to the climate system Agric For Meteorol 2013 169 156 173

[B52] Beven KJ Kirkby MJ A physically based, variable contributing area model of basin hydrology/Un modèle à base physique de zone d“appel variable de l”hydrologie du bassin versant Hydrolog Sci J 1979 24 43 69

[B53] Burkett-Cadena ND McClure CJW Estep LK Eubanks MD Hosts or habitats: What drives the spatial distribution of mosquitoes? Ecosphere 2013 4 1 16

[B54] Leathwick JR Elith J Francis MP Hastie T Taylor P Variation in demersal fish species richness in the oceans surrounding New Zealand: an analysis using boosted regression trees Mar Ecol Prog Ser 2006 321 267 281

[B55] Syfert MM Smith MJ Coomes DA The effects of sampling bias and model complexity on the predictive performance of MaxEnt species distribution models PLoS One 2013 8 e55158 10.1371/journal.pone.0055158 23457462 PMC3573023

[B56] Kadmon R Farber O Danin A Effect of roadside bias on the accuracy of predictive maps produced by bioclimatic models Ecol Appl 2004 14 401 413 10.1890/02-5364

[B57] Walter Reed Biosystematics Unit Mosquito Occurrence Dataset. Smithsonian Institution Accessed via http://www.gbif.org/dataset/88e38292-f762-11e1-a439-00145eb45e9a on 2013-07-26

[B58] Pecoraro H Day H Reineke R Stevens N Climatic and landscape correlates for potential West Nile virus mosquito vectors in the Seattle region J Vector Ecol 2007 32 22 28 10.3376/1081-1710(2007)32[22:CALCFP]2.0.CO;2 17633422

[B59] Elith J Leathwick JR Hastie T A working guide to boosted regression trees J Anim Ecol 2008 77 802 813 10.1111/j.1365-2656.2008.01390.x 18397250

[B60] Hijmans RJ Raster: Geographic Data Analysis and Modeling 2012 R package version 2.1-49

[B61] Phillips SJ Anderson RP Schapire RE Maximum entropy modeling of species geographic distributions Ecol Model 2006 190 231 259 10.1016/j.ecolmodel.2005.03.026

[B62] Ahmed Al AM Badjah-Hadj-Ahmed A-Y Othman Al ZA Sallam MF Identification of wild collected mosquito vectors of diseases using gas chromatography-mass spectrometry in Jazan Province, Saudi Arabia J Mass Spectrom 2013 48 1170 1177 10.1002/jms.3282 24259205

[B63] Ahmed AM Shaalan EA Aboul-Soud MAM Tripet F Al-Khedhairy AA Mosquito vectors survey in the AL-Ahsaa district of eastern Saudi Arabia J Insect Sci 2011 11 176 22958070 10.1673/031.011.17601 PMC3462400

[B64] Khater EI Sowilem MM Sallam MF Alahmed AM Ecology and habitat characterization of mosquitoes in Saudi Arabia Trop Biomed 2013 3 409 427 24189671

[B65] Arishi H Ageel A Rahman MA Hazmi AA Arishi AR Ayoola B Menon C Ashraf J Frogusin O Sawwan F Al-Hazmi M As-Sharif A Al-Sayed M Ageel AR Alrajhi ARA Al-Hedaithy MA Fatani A Sahaly A Ghelani A Al-Basam T Turkistani A Al-Hamadan N Mishkas A Al-Jeffri MH Al-Mazroa YY Alamri MMAEA Outbreak of Rift Valley fever - Saudi Arabia, August-October, 2000 Morb Mortal Wkly Rep 2000 49 905 908 11043643

[B66] Davies FG Risk of a rift valley fever epidemic at the haj in Mecca, Saudi Arabia Rev Off Int Epizoot 2006 25 137 147 16796043 10.20506/rst.25.1.1648

[B67] Zaki A Perera D Jahan SS Cardosa MJ Phylogeny of dengue viruses circulating in Jeddah, Saudi Arabia: 1994 to 2006 Trop Med Int Health 2008 13 584 592 10.1111/j.1365-3156.2008.02037.x 18248565

[B68] Elith J Graham CH Do they? How do they? Why do they differ? On finding reasons for differing performances of species distribution models Ecography 2009 32 66 77 10.1111/j.1600-0587.2008.05505.x

[B69] Diuk-Wasser MA Touré MB Dolo G Bagayoko M Sogoba N Sissoko I Traoré SF Taylor CE Effect of rice cultivation patterns on malaria vector abundance in rice-growing villages in Mali Am J Trop Med Hyg 2007 76 869 874 17488907 PMC1945821

[B70] Knudsen AB Slooff R Vector-borne disease problems in rapid urbanization: new approaches to vector control B World Health Organ 1992 70 1 6 PMC2393336 1568273

[B71] Hassan AN Nogoumy NE Kassem HA Characterization of landscape features associated with mosquito breeding in urban Cairo using remote sensing Egypt J Remote Sens Space Sci 2013 16 63 69

[B72] Hassan AN Beck LR Dister S Prediction of villages at risk for filariasis transmission in the Nile Delta using remote sensing and geographic information system technologies J Egypt Soc Parasitol 1998 28 75 87 9617045

[B73] Sithiprasasna R Linthicum KJ Liu G-J Jones JW Singhasivanon P Use of GIS-based spatial modeling approach to characterize the spatial patterns of malaria mosquito vector breeding habitats in northwestern Thailand Southeast Asian J Trop Med Public Health 2003 34 517 528 15115121

[B74] Cohen JM Ernst KC Lindblade KA Vulule JM John CC Wilson ML Local topographic wetness indices predict household malaria risk better than land-use andland-cover in the western Kenya highlands Malar J 2010 9 328 10.1186/1475-2875-9-328 21080943 PMC2993734

[B75] Moss WJ Hamapumbu H Kobayashi T Shields T Kamanga A Clennon J Mharakurwa S Thuma PE Glass G A dynamic model of some malaria-transmitting anopheline mosquitoes of the Afrotropical region. II. Validation of species distribution and seasonal variations Malar J 2011 10 163 10.1186/1475-2875-10-163 21663661 PMC3123248

