# Supplementary material for: Modeling the distribution of the West Nile and Rift Valley Fever vector Culex pipiens in arid and semi-arid regions of the Middle East and North Africa
Source: Parasit Vectors. 2014 Jun 24;7:289. doi: 10.1186/1756-3305-7-289 (PMC4077837; doi:10.1186/1756-3305-7-289)
Supplement: Additional file 1 — Additional parameters used for generation and evaluation of species distribution models. [file 1756-3305-7-289-S1.doc]

**Additional file 2**

**Additional methods: Cross validation**

The data set is divided into 10 subsets, from these subsets 10 training sets are created, each excluding a single subset. Boosted regression trees are "grown" for each of the 10 training sets. For each of these 10 boosted regression trees, the predictive error is measured at tree sizes of N=1 trees to N=5000 trees, using the excluded subset as the independent test data. At each value of N, the predictive error values for each of the 10 boosted regression trees are pooled, and the tree size N with the minimum predictive error, N_min_, is the optimal tree size. The final boosted regression tree is then grown from the whole data set, to a size of N_min_ trees.

**Addition Methods: Generating background points from the bias file:**

Geographical sampling bias can occur when samples are collected opportunistically. Sampling effort may be greater in conveniently accessed locations, such as populated areas, near roads or rivers, or in habitat already known to support the species of interest. These locations may not reflect the true range of the species environmental niche, and without correction SDMs trained on these samples will model the distribution of sampling effort, rather than the species of interest. To correct for this bias in our models, we selected background points with the same geographical sampling bias as the presence data used to train the model. To generate an estimate of sampling bias, all mosquito survey records not positive for *Cx. pipiens* (n=632) were categorized as “background”, we considered the distribution of these points to represent general sampling effort. A map of sampling intensity was created from these background points using the kernel density estimator tool in ArcGIS’s Spatial Analyst. This bias surface was used for the selection of pseudo-absence points in the subsequent models.

For BRT models, 10,000 background locations were generated from the bias file using the “genrandompnts “ function in GeoSpatial Modeling Environment [1] which creates randomized points based on a probability surface. The Maxent program independently draws 10,000 random points, weighted on the same bias file, to create the background distribution.

**Additional Methods: Details on common model evaluation parameters**

**Maxent model evaluation: Null Model, AICc, omission rate**

Area under the Receiver Operating Characteristic curve (AUC) is a ranked based metric that measures the probability that a model will rank a presence site higher than a random background site[2]. To test the significance of our Maxent SDM, we generated a null distribution of expected AUC values[3]. Using the program ENMTools[4] , we randomly drew 100 pseudo-datasets of 322 localities from the same biased distribution as the real data. We built Maxent models using these pseudo-datasets to generate a distribution of AUC values against which we tested the observed Maxent AUC for significance[5].

The corrected Akaike information criterion (AICc) was used to measure the tradeoff between goodness of fit and model complexity. To obtain an estimate of the AICc we used ENMTools, which bases AIC calculations on the raw and lambda output of the Maxent model.

Maxent estimates the omission rate of its models given a set of test points and a threshold for classification of the continuous output into binary predictions. Omission rates for our Maxenet models were based on the threshold at which model sensitivity equals its specificity[6].

**BRT Model Evaluation: Pearson correlation, deviance**

BRT models were evaluated using AUC, point biserial correlation (the Pearson correlation coefficient, COR, between model predictions and species presence), and the Bernoulli deviance. BRT assesses the accuracy of predictions using cross-validation, testing predicted values against a subset of the training data withheld during model construction. The cross-validated values reported represent the mean values for each of the 10 test subsets.

**Model comparison: Point biserial correlation and deviance**

Because Maxent and BRT used different test points for their intrinsic measures of model accuracy, to compare accuracy between modeling methods, we calculated the point biserial correlation for each model at a standard set of test points[7]. Calculated as the Pearson correlation coefficient between model predictions and presence/absence test data, it describes how closely large differences in prediction values correspond to large differences in the probability of presence[8].The standard set of test data consisted of 79 presence locations taken from the independent data set, as well as 79 background locations generated using the same sampling bias as the training data (Appendix 1 Fig. 1).

**Model comparison: Agreement between BRT and Maxent output**

We used Pearson's correlation coefficient to measure how closely predictions from models created with Maxent agreed with predictions made using boosted regression. We tested the correlation at two scales to examine if agreement between methods suffered when models were required to extrapolate. The first scale, the training region, consisted of the same 158 test points used to calculate the point biserial correlation. Because these points were sampled from the same region as the training data, and half are occupancy sites, tests at this scale evaluated how well model predictions agree in habitat that is known to be suitable, and with very little extrapolation. We also selected another 158 random points, sampled with equal probability from anywhere in the model extent (Appendix 1 Figure 2), and referred to this as the "expanded region". Comparisons at this scale test how well the models agree in areas with a smaller proportion of suitable habitat, and when extrapolating beyond the training environment.

1. Beyer HL: **Geospatial Modeling Environment (Version 0.7.2.1)**. (software). URL: http://www.spatialecology.com/gme. 2012.

2. Phillips SJ, Anderson RP, Schapire RE: **Maximum entropy modeling of species geographic distributions**. *Ecol Model* 2006, **190**:231–259.

3. Raes N, Steege ter H: **A null-model for significance testing of presence-only species distribution models**. *Ecography* 2007, **30**:727–736.

4. Warren DL, Glor RE, Turelli M: **ENMTools: a toolbox for comparative studies of environmental niche models**. *Ecography* 2010.

5. Merckx B, Steyaert M, Vanreusel A, Vincx M, Vanaverbeke J: **Ecological Modelling**. *Ecol Model* 2011, **222**:588–597.

6. Liu C, Berry PM, Dawson TP, Pearson RG: **Selecting thresholds of occurrence in the prediction of species distributions**. *Ecography* 2005, **28**:385–393.

7. Zheng B, Agresti A: **Summarizing the predictive power of a generalized linear model.** *Stat Med* 2000, **19**:1771–1781.

8. Phillips SJ, Dudík M: **Modeling of species distributions with Maxent: new extensions and a comprehensive evaluation**. *Ecography* 2008, **31**:161–175.
