# Supplementary material for: Modeling the distribution of the West Nile and Rift Valley Fever vector Culex pipiens in arid and semi-arid regions of the Middle East and North Africa
Source: Parasit Vectors. 2014 Jun 24;7:289. doi: 10.1186/1756-3305-7-289 (PMC4077837; doi:10.1186/1756-3305-7-289)
Supplement: Additional file 2 — Supplemental maps of background points and additional Maxent output. [file 1756-3305-7-289-S2.docx]

Additional file 1: Figure S1


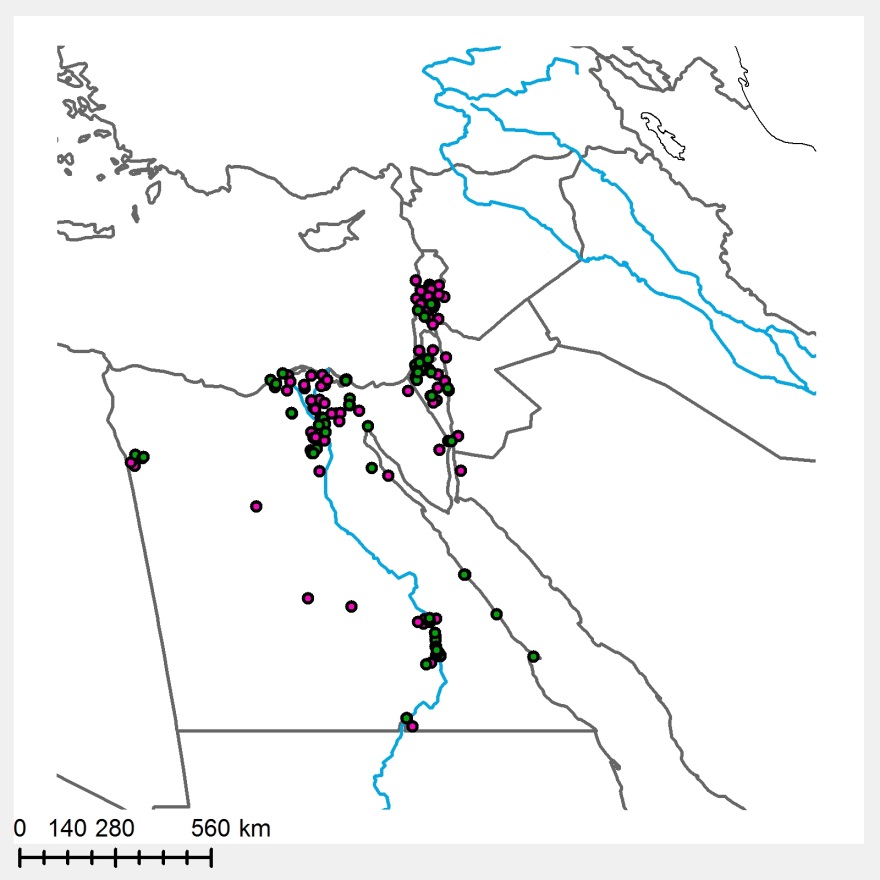


LEGEND Additional file 1: Figure S1

Locations of sampling points for testing model accuracy in the “Training Region”. Green points (N=79) are occurrence records from the Walter Reed Mosquito Occurrence Data Set. Pink points (N=79) are background points drawn from the same distribution of sampling effort used to train the model.

Additional file 1: Figure S2


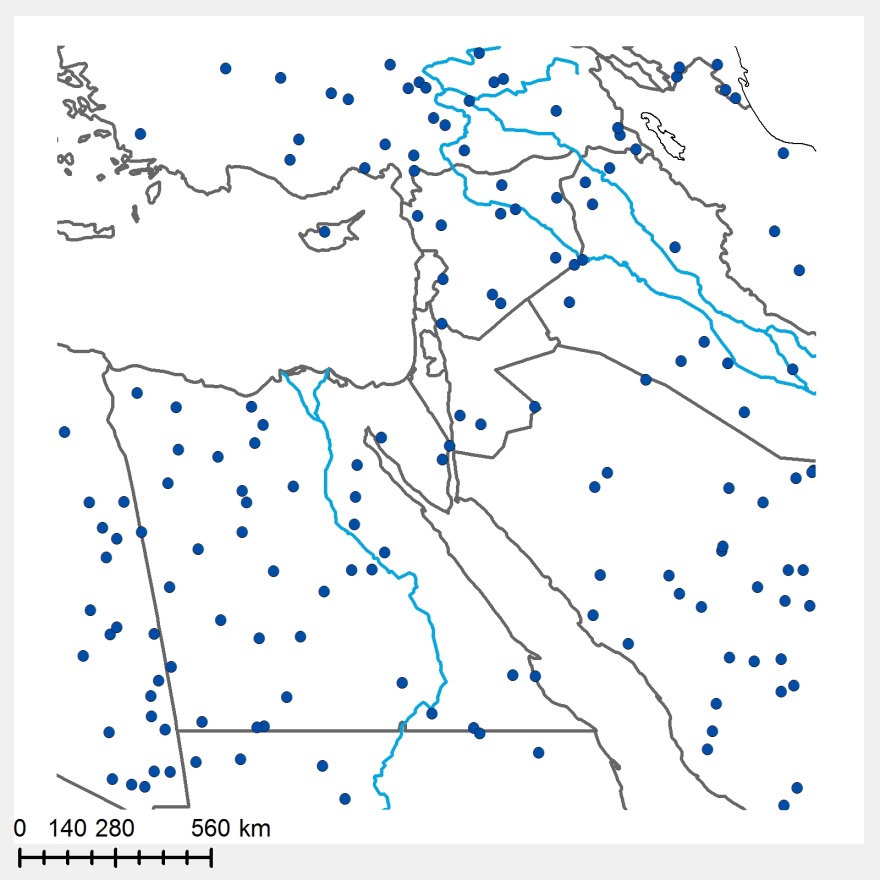


LEGEND Additional file 1: Figure S2

Locations of sampling points (N=158) for testing model accuracy in the “Expanded Region”. Points were randomly sampled from the entire extent of model predictions.

Additional file 1: Figure S3


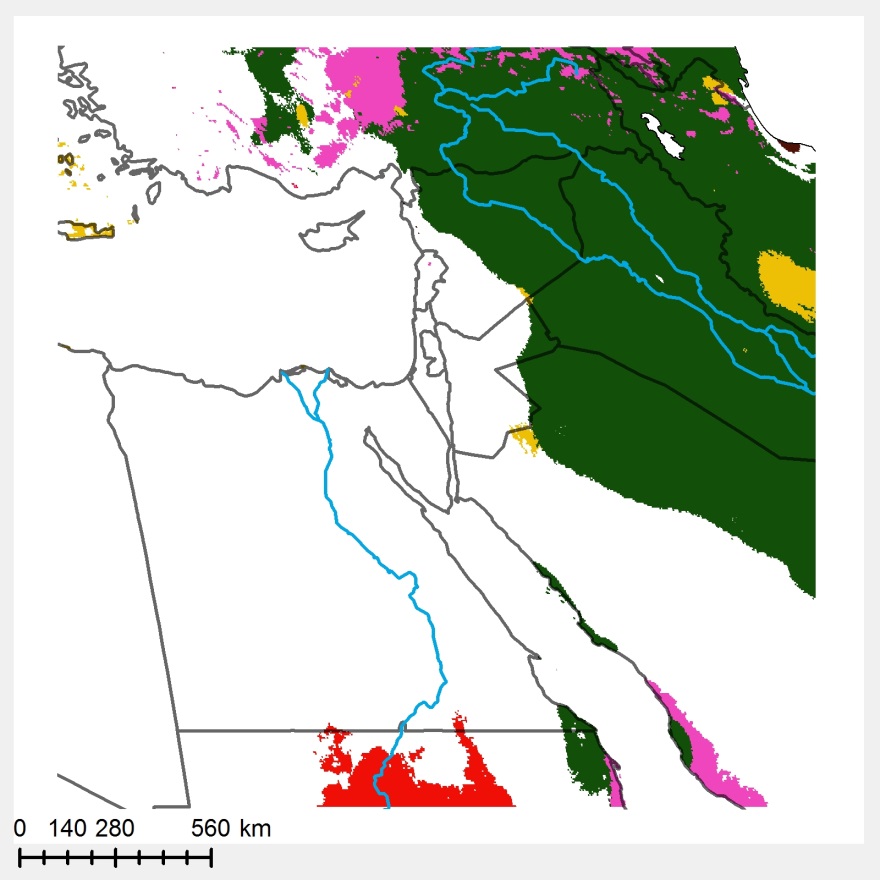


LEGEND Additional file 1: Figure S3

Identity of and distribution of environmental features with values that most exceed those of the training range.

Additional file 1: Figure S4


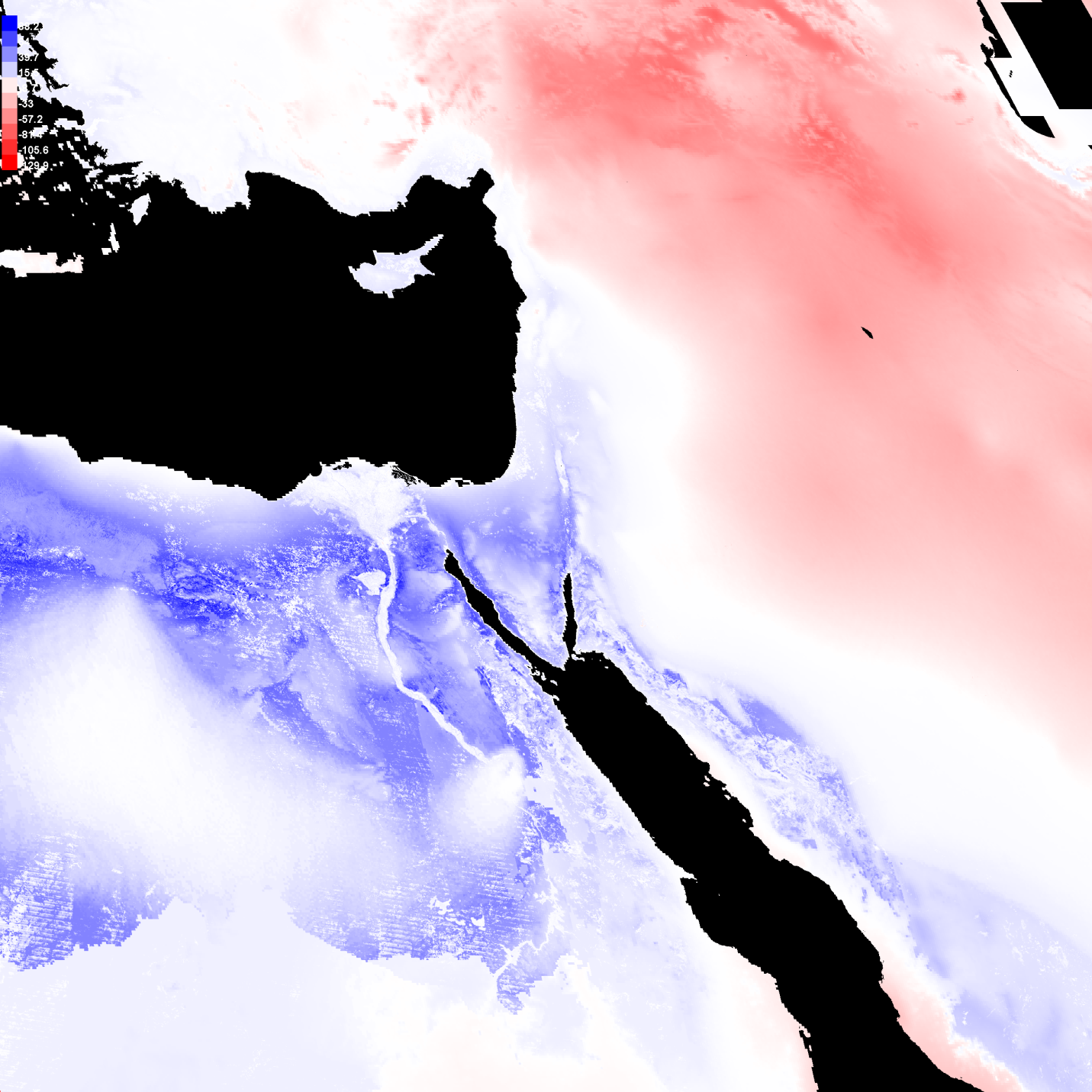


Additional file 1: Figure S4 LEGEND

Multivariate environmental similarity surface (MESS) for MaxentPop model. The MESS analysis indicates the similarity between the environmental variables used in model predictions, and the data used in model training. Areas in red have one or more variables outside the range used in the training data, so predictions in these areas should be interpreted cautiously.
